# The divergent roles of tgf-β isoforms in thyroid cancer and goiter: from mechanistic insights to clinical biomarker potential in thyroidology

**DOI:** 10.1016/j.clinsp.2025.100800

**Published:** 2025-10-15

**Authors:** Demet Sengul, Ilker Sengul, José Maria Soares Júnior

**Affiliations:** a Giresun University, Faculty of Medicine, Department of Pathology, Giresun, Turkey; b Giresun University, Faculty of Medicine, Thyroidology Unit, Giresun, Turkey; c Giresun University, Faculty of Medicine, Division of Endocrine Surgery, Giresun, Turkey; d Giresun University, Faculty of Medicine, Department of General Surgery, Giresun, Turkey; e Disciplina de Ginecologia, Departamento de Obstetrícia e Ginecologia, Laboratório de Ginecologia Estrutural e Molecular (LIM-58), Faculdade de Medicina, Hospital das Clínicas, Universidade de São Paulo, São Paulo, SP, Brazil

## Abstract

• TGF-β isoform levels show divergent perioperative dynamics in thyroid cancer versus goiter.
• The pro-tumorigenic role of TGF-β1 (via EMT) is contrasted with TGF-β3′s potential anti-fibrotic function.
• Pre-operative TGF-β1 may predict radioiodine resistance in DTC (HR=2.3), suggesting prognostic utility.
• TGF-β2 is associated with reduced goiter recurrence, highlighting its biomarker potential.
• Multicenter studies with standardized assays are urgently needed to validate clinical thresholds.

Thyroidology remains sought to investigate the attitude of accurate preoperative diagnosis of Differentiated Thyroid Carcinoma (DTC) by using various modalities, such as Transforming Growth Factor-Beta (TGF-β), a multifunctional cytokine that possesses a delicate balance between replication and cell death in the delicate gland.^1^, ^2^, ^3^, ^4^, ^5^, ^6^, ^7^, ^8^, ^9^, ^10^, ^11^, ^12^ We read with great interest regarding the article entitled “Transforming Growth Factor-Beta (TGF-β) Dynamics in Thyroid Pathologies: A Comprehensive Analysis of Pre- and Post-Surgery Levels in Differentiated Thyroid Cancer and Nodular Goiter”. Bednarczyk and colleagues^1^ reported valuable insights into the perioperative changes of TGF-β1, −2, and −3 for thyroidologists. The inclusion of multiple TGF-β isoforms and the comparative analysis across different diagnostic categories and surgical time points represent a significant strength of the research. The authors well-articulated their purpose to contribute to the understanding of TGF-β1′s role by assessing the pre- and postoperative concentrations of TGF-β1–3 cytokines in nodular goiter. While the role of TGF-β1 has been explored (Table 1), a comparative analysis of all three isoforms (TGF-β1, -β2, -β3) in the perioperative setting, and their distinct mechanistic roles in DTC versus goiter, remains lacking and is crucial for their development as clinical biomarkers.

**Table 1:** Proposed divergent roles of TGF-β isoforms in thyroid pathologies.

Isoform	Proposed Role in Differentiated Thyroid Carcinoma, DTC	Proposed Role in Nodular Goiter	Key References
TGF-1	Pro-tumorigenic, promotes Epithelial-Mesenchymal Transition, EMT, via Smad3 phosphorylation, predicts radioiodine resistance.	Pro-fibrotic, involved in inflammation.	Wang et al.^15^
TGF-2	Potential pro-tumorigenic/variable, data sparser.	Associated with reduced recurrence	Loh et al.^13^
TGF-3	May counteract Epithelial-Mesenchymal Transition, EMT, by upregulating E-cadherin, potential anti-tumorigenic role.	Anti-fibrotic, mediates inflammation resolution.	Sisto et al.^14^

However, addressing the following points might further enhance the rigor and impact of this study:

i) Sample Size Considerations: The relatively small number of cases in the DTC and hyperactive nodular goiter groups compared to the neutral nodular goiter group warrants further discussion. As such, elaborating on how this discrepancy might affect the statistical power and generalizability of the outcomes, particularly for the smaller subgroups. For a broader context, referencing larger meta-analyses would powerfully emphasize that while Bednarczyk et al.'s^1^ study provides valuable insights into TGF-β dynamics, the smaller subgroups necessitate validation through larger cohort studies, especially for TGF-β2 and -β3, where data are currently sparser.
ii) Balancing Focus Across TGF-β Isoforms: While the study measures TGF-β1, −2, and −3, the abstract and conclusions appear to emphasize TGF-β1 primarily. Given that the results section provides data for all three isoforms, the discussion would provide a more comprehensive interpretation of the findings related to TGF-β2 and TGF-β3 and the rationale for the predominant focus on TGF-β1 within the broader context of thyroid pathologies. This is especially pertinent considering their potentially divergent functions. Integrating studies demonstrating TGF-β3’s anti-fibrotic role in goiter could offer a more balanced perspective on the distinct contributions of each isoform (Table 1).
iii) In-depth Interpretation of Results: The results section clearly outlines the observed changes in TGF-β levels before and after surgery. The observed postoperative rise in TGF-β1, −2, and −3 in DTC aligns with its known pro-tumorigenic role, particularly through the induction of Epithelial-Mesenchymal Transition (EMT), wherein TGF-β1 promotes EMT via Smad3 phosphorylation. Interestingly, TGF-β3 may counteract this process by upregulating E-cadherin, suggesting a complex, isoform-specific interplay.^13^ Conversely, the decline in TGF-β1 and -β2 in nodular goiter after surgery likely reflects a resolution of the inflammatory and fibrotic milieu, a process potentially mediated by TGF-β3′s anti-fibrotic effects.^14^ This stark dichotomy underscores the critical need for isoform-specific assays in clinical practice. For instance, the correlation between high preoperative TGF-β1 levels and radioiodine resistance in DTC (HR=2.3, *p* = 0.01) suggests its utility as a prognostic biomarker to guide adjuvant therapy decisions.^15^ Similarly, the association of TGF-β2 with reduced goiter recurrence highlights its potential predictive value (Table 1). Future studies must therefore focus on validating these isoform-specific thresholds using standardized, robust assay platforms to mitigate variability and pave the way for clinical application.
iv) Contextualization with Existing Literature: While you acknowledge previous research on TGF-β1 in thyroid diseases, a more thorough comparison of your findings with the existing body of literature would provide a richer context for your results. This could include a more detailed discussion of studies that have examined perioperative TGF-β changes and the specific roles of TGF-β1, −2, and −3 in different thyroid pathologies. For instance, studies indicating higher TGF-β1 expression in thyroid cancers could be further discussed in relation to your pre- and post-operative findings in DTC patients. Furthermore, contrasting Bednarczyk et al.’s^1^ findings with conflicting reports that found no significant perioperative TGF-β changes in DTC, potentially attributing this to tumor heterogeneity or subtle methodological differences, would strengthen the argument for the critical need for multicenter collaborations and the harmonization of research protocols to ensure robust and generalizable results in future studies.
v) Technical Considerations: A crucial note on methodological variability is warranted. TGF-β measurements are influenced by sample handling. Bednarczyk et al.1 stated that their blood samples were collected into heparinized vials before and after surgery, immediately transferred to the laboratory for centrifugation and storage at −80 °C, and thawed immediately before the Bio-Plex assay. While these steps indicate careful handling, recognizing the sensitivity of TGF-β measurements, particularly TGF-β1, it is essential to highlight that pre-analytical factors, such as platelet contamination, can artificially elevate TGF-β1 levels, necessitating adherence to guidelines from reputable organizations, such as the Journal of Immunological Methods, on pre-analytical factors.

In essence, the work by Bednarczyk et al.^1^ provides a valuable springboard for a more nuanced understanding of TGF-β biology in thyroid disorders. Our analysis underscores that the perioperative dynamics of TGF-β are not monolithic but are profoundly isoform-specific and pathology-dependent. The rise in TGF-β1–3 post-thyroidectomy in DTC likely reflects a pro-tumorigenic shift, whereas its decline in goiter suggests inflammation resolution, potentially mediated by TGF-β3. Critically, these patterns hold significant translational promise. The association of TGF-β1 with radioiodine resistance and TGF-β2 with reduced recurrence positions them as compelling candidates for prognostic and predictive biomarkers. However, unlocking this clinical potential is contingent upon overcoming key limitations: validating these findings in larger, prospective cohorts and standardizing pre-analytical and analytical methodologies to ensure reliability. Therefore, this commentary serves not only as a critique but as a strategic roadmap in thyroidology. Future research must prioritize multi-institutional collaborations to define clinically actionable, isoform-specific thresholds, ultimately paving the way for integrating TGF-β profiling into personalized management strategies for patients with thyroid nodules and cancer for thyroidologists.

## Declaration of competing interest

The authors declare no conflicts of interest.
